# Static and dynamic changes of intrinsic brain local connectivity in internet gaming disorder

**DOI:** 10.1186/s12888-023-05009-y

**Published:** 2023-08-09

**Authors:** Xiaoyu Niu, Xinyu Gao, Mengzhe Zhang, Jinghan Dang, Jieping Sun, Yan Lang, Weijian Wang, Yarui Wei, Jingliang Cheng, Shaoqiang Han, Yong Zhang

**Affiliations:** 1 Department of Magnetic Resonance Imaging, First Affiliated Hospital of Zhengzhou University, Zhengzhou, Henan China; 2 Key Laboratory for functional magnetic resonance imaging and molecular imaging of Henan Province, Henan Province, China; 3 Department of Psychiatry, First Affiliated Hospital of Zhengzhou University, Zhengzhou, China

**Keywords:** Internet gaming disorder (IGD), Dynamic, Intrinsic local connectivity, Regional homogeneity (ReHo), Resting-state functional magnetic resonance imaging (rs-fMRI)

## Abstract

**Background:**

Studies have revealed that intrinsic neural activity varies over time. However, the temporal variability of brain local connectivity in internet gaming disorder (IGD) remains unknown. The purpose of this study was to explore the alterations of static and dynamic intrinsic brain local connectivity in IGD and whether the changes were associated with clinical characteristics of IGD.

**Methods:**

Resting-state functional magnetic resonance imaging (rs-fMRI) scans were performed on 36 individuals with IGD (IGDs) and 44 healthy controls (HCs) matched for age, gender and years of education. The static regional homogeneity (sReHo) and dynamic ReHo (dReHo) were calculated and compared between two groups to detect the alterations of intrinsic brain local connectivity in IGD. The Internet Addiction Test (IAT) and the Pittsburgh Sleep Quality Index (PSQI) were used to evaluate the severity of online gaming addiction and sleep quality, respectively. Pearson correlation analysis was used to evaluate the relationship between brain regions with altered sReHo and dReHo and IAT and PSQI scores. Receiver operating characteristic (ROC) curve analysis was used to reveal the potential capacity of the sReHo and dReHo metrics to distinguish IGDs from HCs.

**Results:**

Compared with HCs, IGDs showed both increased static and dynamic intrinsic local connectivity in bilateral medial superior frontal gyrus (mSFG), superior frontal gyrus (SFG), and supplementary motor area (SMA). Increased dReHo in the left putamen, pallidum, caudate nucleus and bilateral thalamus were also observed. ROC curve analysis showed that the brain regions with altered sReHo and dReHo could distinguish individuals with IGD from HCs. Moreover, the sReHo values in the left mSFG and SMA as well as dReHo values in the left SMA were positively correlated with IAT scores. The dReHo values in the left caudate nucleus were negatively correlated with PSQI scores.

**Conclusions:**

These results showed impaired intrinsic local connectivity in frontostriatothalamic circuitry in individuals with IGD, which may provide new insights into the underlying neuropathological mechanisms of IGD. Besides, dynamic changes of intrinsic local connectivity in caudate nucleus may be a potential neurobiological marker linking IGD and sleep quality.

**Supplementary Information:**

The online version contains supplementary material available at 10.1186/s12888-023-05009-y.

## Introduction

Internet gaming disorder (IGD) is a behavioral addiction that involves excessive involvement with online games despite knowing the negative consequences, characterized by impaired executive control, excessive reward-seeking and persistent cravings[1, 2]. It has been linked to a variety of negative consequences such as weak time management skills, poor academic or work performance, physical and psychological disorders and social deficiencies[3–5]. Given its growing prevalence and severe adverse effects, a description of IGD has been included in the Diagnostic and Statistical Manual of Mental Disorders, Fifth Edition (DSM-5), Sect. 3, and in the International Classification of Diseases, 11th Edition (ICD-11)[6]. Elucidating the neurobiological mechanism of IGD is of great significance for determining new therapeutic strategies for IGD.

In recent years, numbers of neuroimaging studies have been devoted to revealing the neuropathological mechanism of IGD. Spontaneous neural activity under resting state is important for understanding neuropathological and neurophysiological conditions[7]. Due to its ease of implementation and the prevention of possible task-related confounds, resting state functional magnetic resonance imaging (rs-fMRI) has become an increasingly popular research modality for studying neuropsychiatric disorders. It can measure low-frequency fluctuations of the blood oxygen level dependent (BOLD) signals to detect spontaneous neural activity. One of the widely used metrics for describing resting-state spontaneous neural activity is the static regional homogeneity (sReHo), which characterizes the synchronization of spontaneous low frequency BOLD signal fluctuations within brain regions by computing Kendall’s coefficient of concordance (KCC). It has been certified to be a reliable metric with high retest reliability[8] and has been widely used to examine abnormal spontaneous neural activity in many neuropsychiatric disorders such as smoking, schizophrenia and major depressive disorder[9–12]. ReHo values represent the degree of local connectivity between given voxels and adjacent voxels. Previous study has found widely higher ReHo in the frontal lobe in IGD subjects in comparison of control subjects, indicating increased cognitive control function-related neural activities[13]. Decreased spontaneous brain activity in the left superior obitofrontal cortex (OFC) and putamen as well as increased OFC-putamen connectivity after the cognitive behavior therapy (CBT) were observed by Han.et al, suggesting that the OFC-striatal circuit may serve as a target for brain-based potential treatments of IGD[14].

There is emerging evidence that the intrinsic neural activity varies over time[15, 16]. The above rs-fMRI studies of IGD were based on the assumption that signals were stationary during scanning and ignored the dynamic changes of spontaneous brain activity in time dimension. The dynamic ReHo (dReHo) can reflect the information of temporal dimension by capturing the temporal variability in regional neural activity synchronization at a shorter time window. It was more sensitive to detect regional neural activity differences between patients and control subjects by using dReHo than traditional sReHo metric[17, 18]. The dynamic metric has been extensively applied in many diseases such as stroke, smoking addiction, anxiety disorder and schizophrenia[19–22]. The calculation of dReHo is based on the sliding window method, which has demonstrated excellent performance in evaluating the temporal variability of regional neural activity[20], and has been extensively used because of its ease of implementation and simplicity[23]. The combination of static and dynamic metrics may provide a more comprehensive understanding of the neuropathological changes of IGD.

Existing data have indicated an association between IGD and sleep quality. Several studies have found that pathological game use reduced sleeping time[24], delayed bedtime[25], and increased drowsiness and fatigue in the daytime[26, 27]. Currently, there were relatively few studies on the relationship between neuroimaging markers of IGD and sleep quality. Zheng and his colleagues have found that the right posterior hippocampus (pHIP)-left caudate resting-state functional connectivity (rs-FC) was correlated with both the IGD and sleep quality and mediated the relationship between the two[28]. However, the relationship between aberrant static and dynamic intrinsic brain local connectivity of IGD and sleep quality has not been elucidated.

Given the time-varying properties of spontaneous neural activity in resting state, the temporal variability of intrinsic brain local connectivity in individuals with IGD and its relationships to clinical characteristics have been unclear. The primary purpose of this study was to explore the patterns of altered intrinsic brain local connectivity in individuals with IGD, combined with sReHo and dReHo metrics. The second purpose was to examine the correlations of these neuroimaging findings with the clinical characteristics of IGD (including online gaming addiction severity and sleep quality). We hypothesized that altered intrinsic brain local connectivity would be observed in individuals with IGD and that these findings might related to online gaming addiction severity and sleep quality.

## Materials and methods

### Participants

A total of 80 subjects recruited from the local hospital (36 with and 44 without IGD) were included in this study. All subjects were right-handed males. This study used the DSM-5 and Young’s Internet Addiction Test (IAT) as the diagnostic criteria for IGD subjects. All subjects were first assessed for IGD diagnosis by an experienced psychiatrist via the DSM-5 diagnostic criteria. The IAT was used to evaluate the severity of online gaming addiction. Among the 20 items on the scale are descriptions of psychological dependence, compulsive Internet usage, withdrawal, problems at work and school, poor time management sleep problems, and family problems caused by Internet use. Each item is graded by 1 to 5 and the total scores range from 1 to 100. The Pittsburgh Sleep Quality Index (PSQI) was used to evaluate the sleep quality. The scores range from 0 to 21, with higher scores indicating worse sleep quality.

Subjects with an IAT score of 50 or greater and meeting five or more DSM-5 based diagnostic criteria for IGD were included in the IGD group[29]. Those who scored no less than 50 on the IAT and did not meet the DSM-5 diagnostic criteria were included in the control group. All participants underwent structured psychiatric interviews (using the Mini-International Neuropsychiatric Interview) performed by an experienced psychiatrist. None of the participants had (i) a history of anxiety, major depression, substance use disorders or attention-deficit hyperactivity disorder; (ii) mental retardation; and (iii) neurological illness or injury. On the day of the scan, it was requested that all participants refrain from using any medications. This study was carried out in accordance with the Declaration of Helsinki and approved by the Local Medical Ethics Committee of the First Affiliated Hospital of Zhengzhou University (2022-KY-0438). Informed consent was obtained from all participants.

### Data acquisition and preprocessing

MRI data were acquired using the 3T Magnetom Prisma MRI scanner (Siemens Healthcare, Erlangen, Germany) with 64 channel head coils. Each participant was asked to lie down flat, close eyes, not to think of anything, breathe quietly, and avoid falling asleep. We used foam pads and earplugs to minimize head movement and canner noise. The following scanning parameters were used to acquire functional images: repetition time (TR)/echo time = 1000/30ms, flip angle = 70°, field of view (FOV) = 220 × 220 mm^2^, voxel size = 2 × 2 × 2.2 mm^3^, slices = 52, slice thickness = 2.2 mm, and 400 volumes in total.

All fMRI images were initially checked for quality, and any incomplete or artifact-filled images were excluded. Functional data were then preprocessed using the Data Processing Assistant for Resting-State fMRI (DPARSF). Several steps were involved in preprocessing: (i) conversion of data formats (DICOM to NIFTI); (ii) removing first 10 volumes considering the instability of the initial rs-fMRI signal; (iii) slice timing; (iv) realignment (excluding subjects with a maximum head motion > 2.5 mm or rotation > 2.5°); (v) spatial normalization into Montreal Neurological Institute (MNI) space by using EPI templates and resampling with 3 × 3 × 3 mm^3^; (vi) removing linear trends and temporally bandpass filtering (0.01–0.08 Hz) to eliminate low-frequency drift and high-frequency noise influences; (vii) regression of 24 head motion parameters, global signals, white matter and cerebrospinal fluid signals[30].

### sReHo and dReHo calculation

The sReHo was computed using KCC based on DPABI software to quantify the similarity of a voxel’s time series to those of its 27 neighbors[31]. The images were *Z*-transformed and spatially smoothed using an 8 mm full-width half maximum Gaussian kernel for statistical analyses.

An analysis of dynamic regional metric was conducted using the Temporal Dynamic Analysis (TDA) toolkit based on the Data Processing and Analysis of Brain Imaging (DPABI). The dReHo was calculated within a temporal window of a specific size and shape by using the sliding window method. There is an expectation that the window size will be small enough to capture high frequency signals and large enough to seize low frequency signals[19, 32]. Traditionally, window-based analyses have been performed in periods as short as 10 s[33] to as long as 180 s[19, 34]. This study used a sliding window of 50 TR (50 s) and a shift step size of 1 TR (1 s)[35]. We also examined the effect with other window lengths (30 TRs and 80 TRs), and they were included in validation analyses. Across n window, we calculated the coefficient of variation (CV) maps of ReHo for each subject. The CV of a voxel *k* was defined as:

$${CV}_{K}=\sqrt{\frac{{\sum }_{t=1}^{n}({x}_{t}-{x}_{mean}{)}^{2}/n}{{x}_{mean}}}$$

where *x*_*t*_ is ReHo score of voxel *k* over time window *t*, *t =* 1, 2, …, *n*; *x*_*mean*_ is mean score of *x*_*t*_ across time window *t*. Subsequently, individual voxel-wise ReHo CV maps were standardized by dividing the mean values of the whole brain and, furthermore, spatially smoothed (using a full-width Gaussian kernel with a half-maximum of 8 mm).

### Statistics analyses

For comparisons between two groups in demographics and clinical characteristics (i.e. age, years of education, IAT scores and PSQI scores), two-sample t tests were performed using IBM SPSS Statistics software (version 26.0). Statistical significance was set at *p* < 0.05. Using the general linear model (GLM) in SPM12 with age, years of education and mean framewise displacement (FD) as covariates, inter-group analyses were examined to determine whether sReHo and dReHo differed between individuals with IGD and HCs (Gaussian random field theory GRF corrected, *P*_voxel_ < 0.005, *P*_cluster_ < 0.05).

In order to examine the associations between the neuroimaging findings and clinical characteristics (including the severity of online gaming addiction and sleep quality) of IGD, we performed Pearson correlation analyses between brain regions with abnormal activity and IAT scores and PSQI scores.

Binary logistic regression was applied to calculate the predictive probability of brain regions with altered sReHo and dReHo separately, and the predictive probability of combined brain regions with altered sReHo and dReHo for the diagnosis of IGD. Receiver operating characteristic (ROC) curves were constructed using the predictive probability as covariates. Areas under the curve (AUCs) were used to evaluate the diagnostic value of the two metrics and the combination of them. An AUC greater than 0.9 suggested excellent diagnostic efficacy. An AUC between 0.7 and 0.9 suggested good diagnostic efficacy. An AUC between 0.5 and 0.7 suggested poor diagnostic efficacy. Finally, an AUC of no more than 0.5 suggested a lack of diagnostic value.

### Validation analyses

We calculated the dReHo values with the window sizes of 30 TRs and 80 TRs, respectively, and compared the differences between two groups to verify the stability of the present results.

## Results

### Demographic and clinical characteristics of participants

**Fig. 1:**
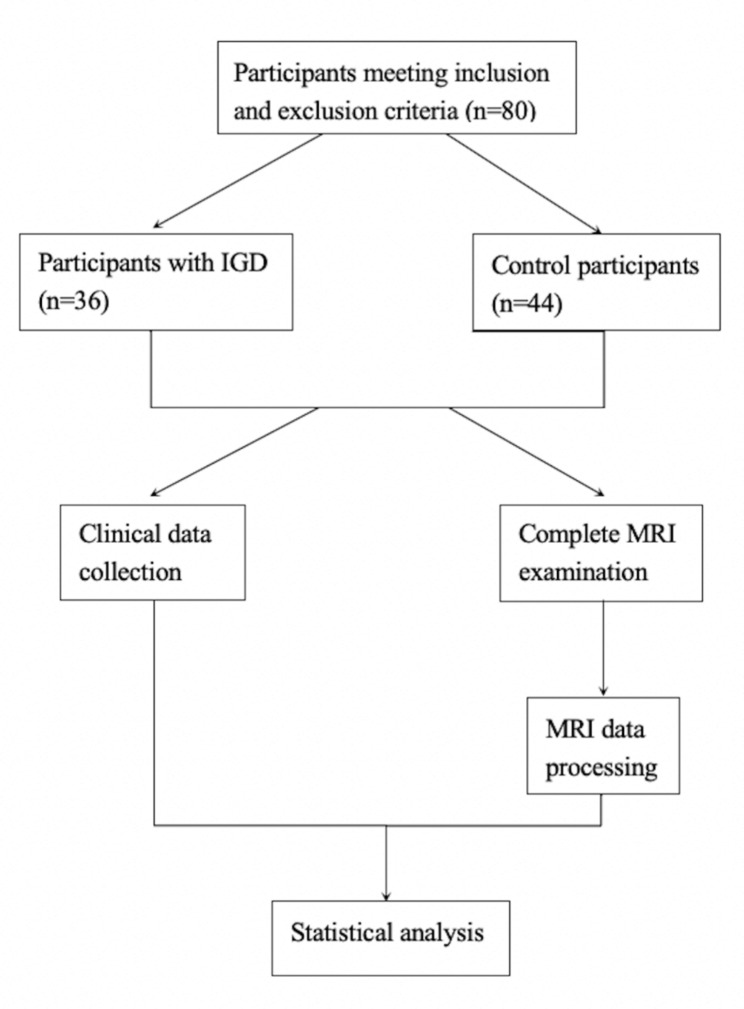
Flow chart of this study

No significant differences were observed in age and years of education between two groups. The IGD group reported significantly higher IAT scores (*t* = 17.94, *p <* 0.001) and PSQI scores (*t* = 3.87, *p <* 0.001) compared with the HC group. Detailed demographic and clinical information were presented in Table 1. The flow chart of this study for demographic and clinical data was showed in Fig. 1.

**Table 1:** Demographic and clinical characteristics of participants

	IGD (36)	HC (44)	*t*	*P* value
Age, years	14.44 ± 2.02	16.11 ± 6.08	-1.71	0.093
years of education	8.75 ± 1.87	9.45 ± 4.39	-0.96	0.339
IAT Score	62.81 ± 10.46	28.34 ± 5.37	17.94	<0.001
PSQI Score	6.83 ± 3.37	4.45 ± 1.52	3.87	< 0.001

Note: Values are presented as mean ± SD; IAT: Internet addiction test; PSQI: Pittsburgh Sleep Quality Index;

IGD: Internet gaming disorder; HC: healthy control

### Differences in sReHo and dReHo between IGDs and HCs

**Fig. 2:**
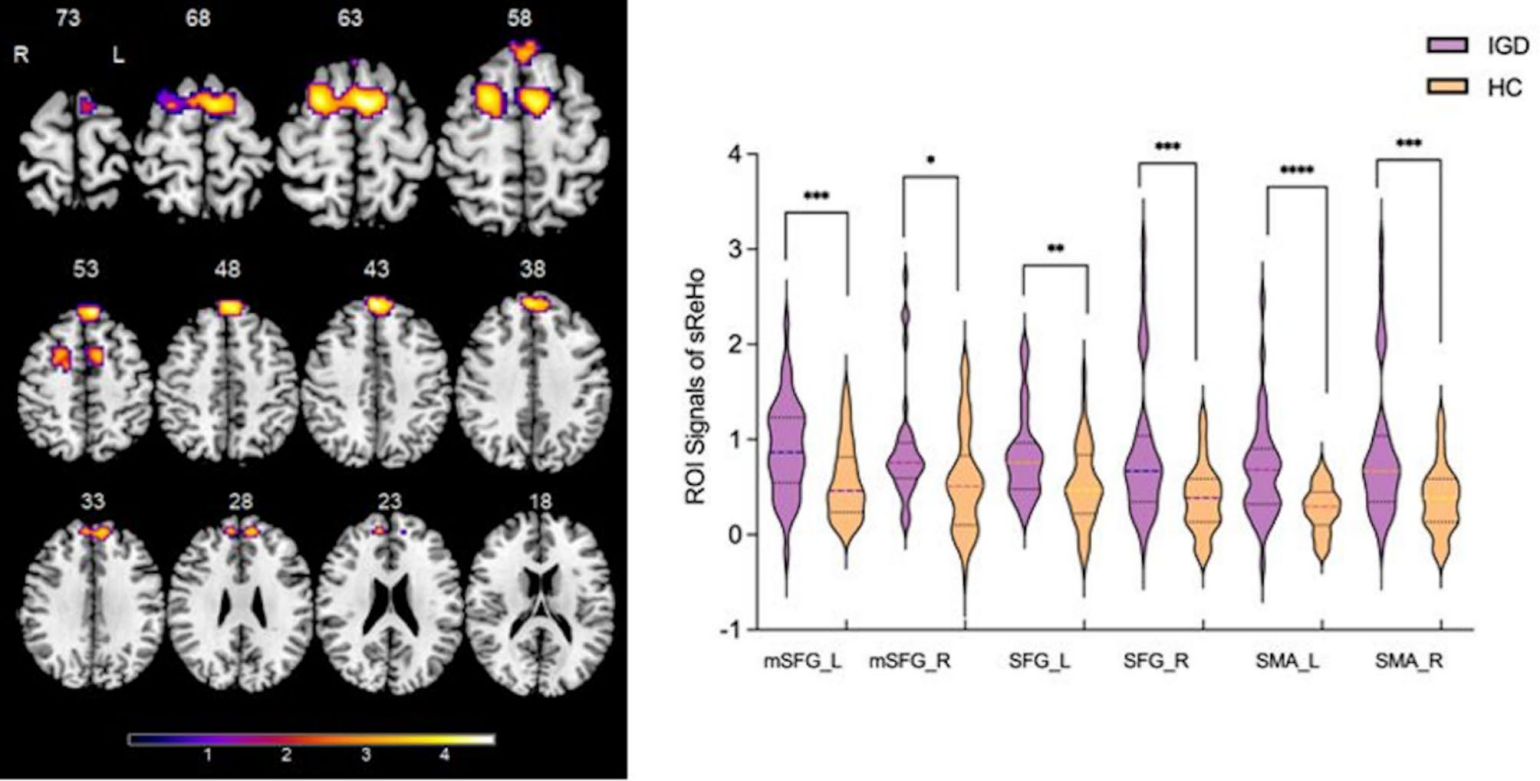
Compared with healthy controls, IGD subjects showed significantly increased static ReHo. * indicates statistically significant differences. Different numbers of * represent different degrees of *P* value. L: left; R: right; mSFG: medial superior frontal gyrus; SFG: superior frontal gyrus; SMA: supplementary motor area

**Fig. 3:**
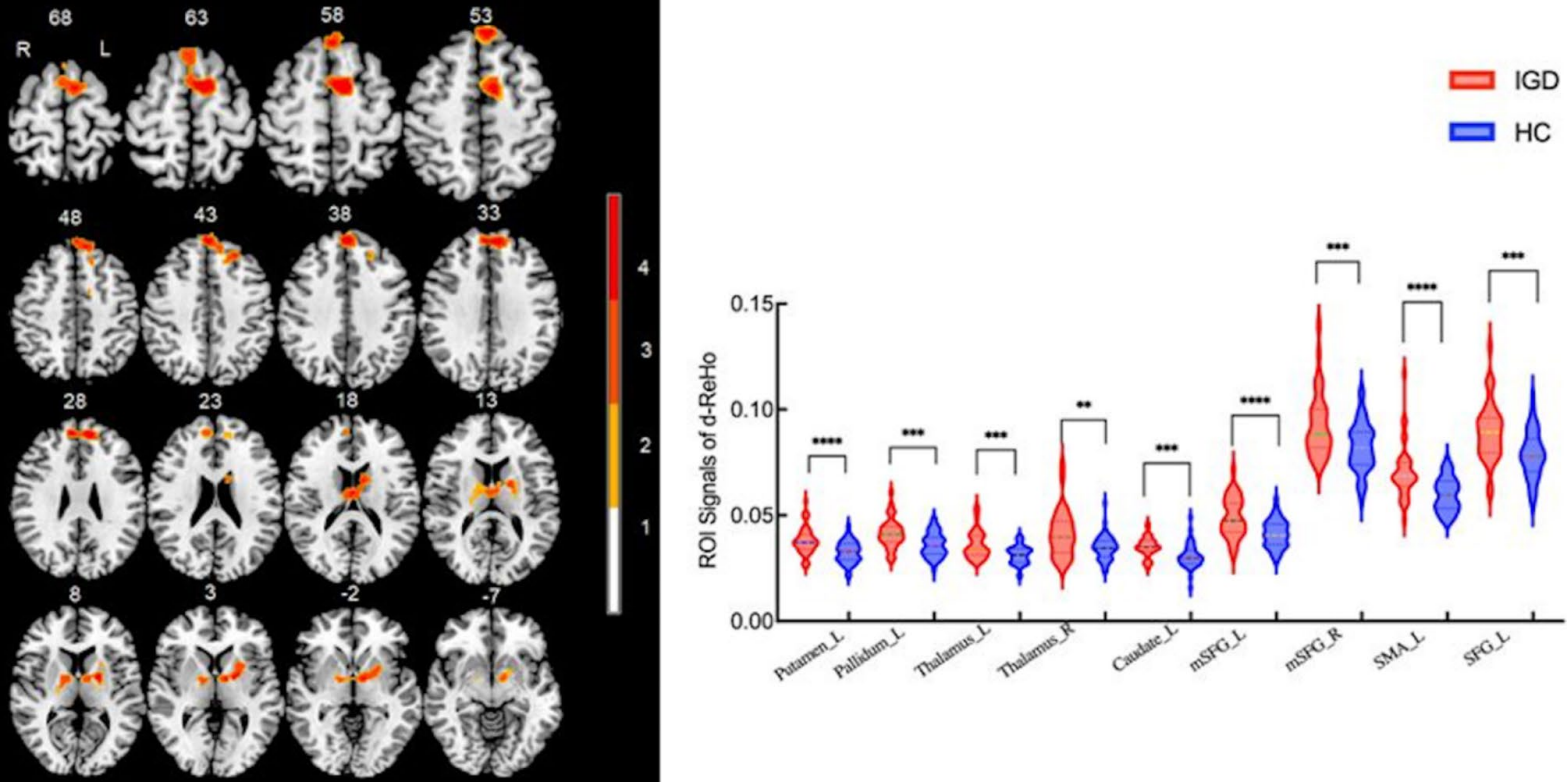
Compared with healthy controls, IGD subjects showed significantly increased dynamic ReHo. * indicates statistically significant differences. Different numbers of * represent different degrees of *P* value. L: left; R: right; mSFG: medial superior frontal gyrus; SMA: supplementary motor area; SFG: superior frontal gyrus

Compared with HCs, IGDs showed significantly increased sReHo in the bilateral medial superior frontal gyrus (mSFG), superior frontal gyrus (SFG), and supplementary motor area (SMA) (Table 2; Fig. 2). Besides, increased dReHo was found in left putamen, left pallidum, bilateral thalamus, left caudate, bilateral mSFG, left SMA and SFG in individuals with IGD compared with HCs (Table 2; Fig. 3).

**Table 2:** Differences of static and dynamic ReHo between IGD and HC groups

Index	Brain Regions	L/R	Peak MNI Coordinates	Number of cluster voxels	*t-*values
			(x,y,z)		
Static ReHo:	mSFG	L	-6,42,54	133	4.61
	mSFG	R	3,51,43	79	4.6
	SFG	L	-12,51,30	65	3.61
	SFG	R	22,7,61	122	4.33
	SMA	L	-6,6,63	139	4.56
	SMA	R	22,7,59	46	4.33
Dynamic ReHo:	Putamen	L	-18, -3,9	69	4.65
	Pallidum	L	-20, -3,7	39	4.15
	Thalamus	L	-12, -4,0	20	3.93
	Thalamus	R	15, -6,6	39	3.44
	Caudate nucleus	L	-9,4,19	27	3.84
	mSFG	L	-3,42,57	143	4.81
	mSFG	R	7,51,28	86	3.92
	SMA	L	-10,2,57	154	4.36
	SFG	L	-13,50,30	118	4

Note: mSFG: medial superior frontal gyrus; SFG: superior frontal gyrus; SMA: supplementary motor area; L: left; R: right; MNI: Montreal Neurological Institute

### Correlation analysis

**Fig. 4:**
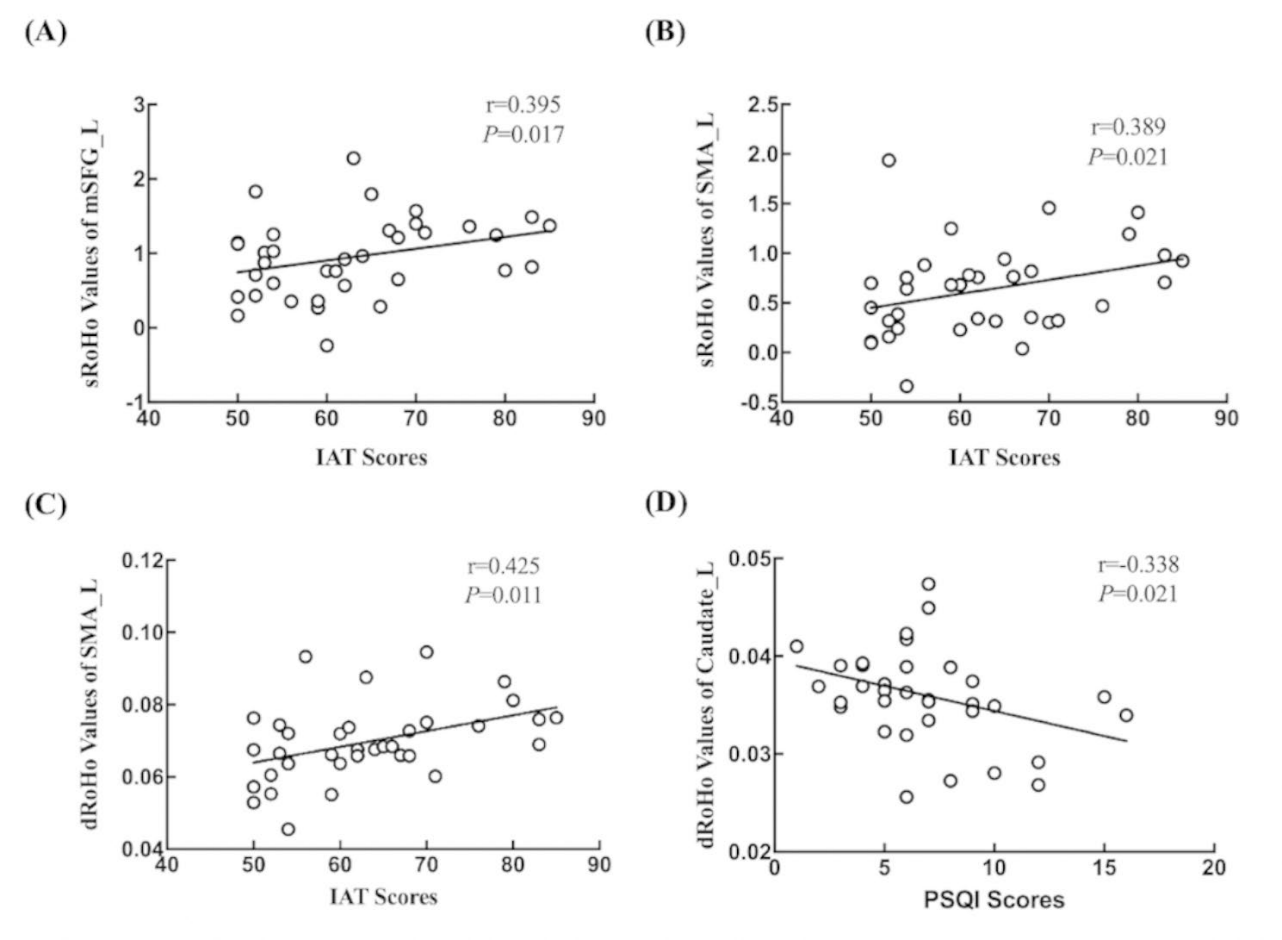
Correlations between sReHo/dReHo values and IAT/PSQI scores in individuals with IGD. (**A**) The sReHo values in the left mSFG were positively correlated with IAT scores. (**B**) The sReHo values in the left SMA were positively correlated with IAT scores. (**C**) The dReHo values in the left SMA were positively correlated with IAT scores. (**D**) The dReHo values in the left caudate nucleus were negatively correlated with PSQI scores. s: static; d: dynamic; ReHo: regional homogeneity; L: left; mSFG: medial superior frontal gyrus; SMA: supplementary motor area

The results showed that the sReHo values in the left mSFG and SMA as well as dReHo values in the left SMA were positively correlated with IAT scores (*r* = 0.395, *p* = 0.017; *r* = 0.389, *p* = 0.021; *r* = 0.425, *p* = 0.011). The dReHo values in the left caudate nucleus were negatively correlated with PSQI scores (*r* = -0.338, *p* = 0.021) **(**Fig. 4**)**.

### ROC curve analysis

**Fig. 5:**
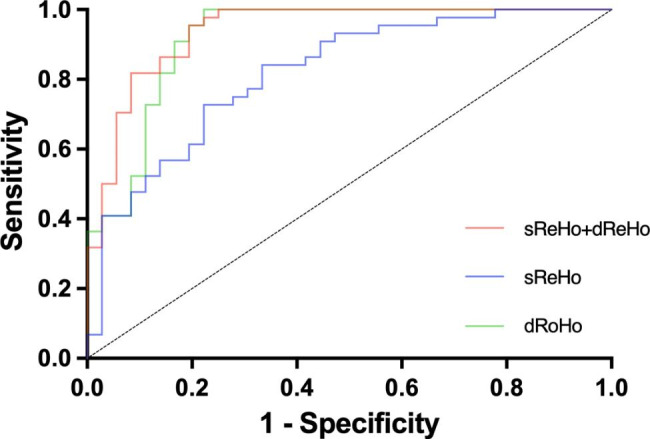
ROC curves of single metric (sReHo or dReHo) and the combination of the two metrics (sReHo + dReHo) for screening IGD in controls. sReHo: static regional homogeneity; dReHo: dynamic regional homogeneity

ROC curves of sReHo and dReHo metrics for IGD screening in controls were constructed based on binary logistic regression. The results showed that the AUCs of brain regions with altered sReHo and dReHo were 0.818 (*P* < 0.001, 95%CI: 0.725 to 0.910) and 0.920 (*P* < 0.001, 95%CI: 0.856 to 0.984), respectively. The AUC of combined sReHo and dReHo was 0.939 (*P* < 0.001, 95%CI: 0.887 to 0.992) (Fig. 5).

### Validation results

As shown in the supplementary materials, the results of dReHo using window size of 30TRs and 80TRs were similar to the present main findings (GRF corrected, *P*_voxel_ < 0.005, *P*_cluster_ < 0.05, Figure S1).

## Discussion

This study showed altered static and dynamic patterns of intrinsic local connectivity in individuals with IGD by incorporating sReHo and dReHo metrics. Relative to HCs, IGD subjects showed both increased static and dynamic intrinsic local connectivity in bilateral mSFG, superior SFG, and SMA. Besides, higher dynamic intrinsic local connectivity in the left dorsal striatum (e.g. putamen, pallidum and caudate nucleus) and bilateral thalamus were reported in IGD in the current study. The above brain regions are important components of the frontostriatothalamic circuitry, which have been shown to play an important role in reward processing and cognitive control. Furthermore, the ROC analysis of the static and dynamic ReHo metrics revealed that intrinsic brain local connectivity had the potential capacity to distinguish individuals with IGD from HCs. Besides, the study also found that dReHo values in the left caudate nucleus displayed a significant correlation with PSQI scores. In general, these findings provided new insights into the neuropathological mechanisms of IGD and shed new light on the neural correlations of IGD and sleep quality.

The frontal cortex, especially the dorsolateral prefrontal cortex (PFC), including SFG and SMA, plays a crucial role in executive functions such as inhibitory control[36–38]. The normal population is equipped with a flexible cognitive control system for making decisions as well as resisting rewards that have negative long-term consequences, indicating that the reward system is regulated from the top down[39]. However, this trait appears to be reversed in addicts, who exhibit a loss of cognitive control over excessive reward-seeking behaviors and cues to addiction. In subjects with IGD, the reward system exerts a robust bottom-up driving effect over the control system, as revealed by a dynamic causal modeling (DCM) analysis[40]. According to several models of IGD, the impairment in executive function and inhibitory control may at the core of IGD[41, 42]. Previous study found that the IGD group was remarkably hyperactive during the No-Go trials in the left mSFG, which may indicate that IGD subjects showed decreased efficiency in response inhibition process and needed greater levels of neuronal involvement[43]. This study found increased intrinsic neural activity in SFG and SMA, and these changes were positively correlated with the IAT scores, which may suggest that the response inhibition system in IGD subjects may be overworked and under greater cognitive stress than in healthy individuals, and that such changes correlate with the severity of online gaming addiction.

The striatum has shown to be involved in reward-based learning, reward processing and reinforcement. Our results focused primarily on the dorsal striatum, including the putamen and the caudate nucleus, where the putamen has been functionally related to the motor and sensory cortex, which is responsible for compulsive behavior and involved in habit formation, and the caudate nucleus receives projections primarily from dorsolateral PFC[44–46]. According to prior animal models, switching control sites from the ventral striatum to the dorsal striatum mediated the transition from initial rewards-driven drug use to compulsive use and habitual dependence[47, 48]. In early stages of addiction, the ventral striatum plays a critical role, but later on, the dorsal striatum may play a greater role in compulsive and habitual behaviors of the disorders[48, 49]. The putamen has shown to be responsible for the transition to habitual stimulus-controlling behavior[47], which may be viewed as a pathological endpoint of addictive disorders. In this study, the increased intrinsic activity of the dorsal striatum (rather than ventral striatum) in the task-free state may indicate that IGD subjects spend a lot of time on online games, which may have shifted from the initial reward-driven behavior to habitual compulsive behavior[50].

In addiction studies, it has been demonstrated that exposure to addictive behaviors increases dopamine levels in the reward circuit, which includes the striatum (e.g. caudate nucleus and putamen) as well as the lateral and medial PFC[51]. The dopamine projection of the midbrain travels from the globus pallidus to the thalamus, then from the thalamus to the frontal lobe, so there is an input and output projection in the thalamus[52]. The thalamus plays a central role in perception integration, cognitive, emotional processing[53], and executive function[54], acting as a relay station between the striatum and the cortex[55]. Individuals with difficulty in emotional regulation usually adopt maladaptive behaviors in order to avoid or relieve negative emotions, which may lead to the development of psychopathology[56, 57]. When experiencing negative emotions, patients with IGD may use online games as a maladaptive strategy to avoid existing problems and gain relief from emotional distress. In addition, the thalamus monitors prefrontal activity through thalamocortical inputs and then integrates this information with signals from the motivational and sensorimotor systems[58]. Alterations in the thalamocortical circuits may impair the integration of motivational and sensorimotor information in IGD patients, thereby compromising their goal-orientation system and making them more dependent on the habitual system. Many previous studies showed that the thalamus had disturbed rs-FC with frontal and striatal regions in IGD[59, 60]. Previous study reported that problem behaviors of IGD may be caused by the mechanisms underlying the negative correlation between reward processing and prefrontal inhibition[61]. Addicted individuals have a hyperactive reward system that drives the individual to further reward-seeking behavior. However, the brain regions responsible for executive control may not be effective in exerting top-down control over the brain’s desire to online games, and such dysregulation may lead to persistent addiction despite knowing negative consequences. Overall, increased intrinsic local connectivity in these regions of IGD subjects indicated the impaired frontostriatothalamic circuitry, which may suggest that uncontrolled online gaming behaviors resulted from the imbalance between executive control and reward processing.

Previous studies have found that individuals with IGD often have sleep problems[62], which was consistent with our findings. The PSQI scores of IGD subjects were higher than that of healthy controls. The caudate nucleus was not only involved in reward processing, but also in sensory processing, hyper-arousal, and sleep regulation[63]. Early animal studies have shown that caudate nucleus lesion can cause behavioral restlessness and hyperreactivity, suggesting inhibitory dysregulation of sensory input[64]. In addition, one study found that individual differences in sleep duration and sleep quality in adolescents were associated with task-induced caudate nucleus activation[65]. The current results showed that dReHo in the right caudate nucleus was negatively correlated with PSQI scores in IGD subjects, which may suggest that temporal variability of neural activity in the caudate nucleus may be a promising neurobiological marker linking IGD and sleep quality.

### Limitation

Several limitations of the study should not be ignored. Firstly, the participants included in the current study were all males. Previous studies have reported gender differences in neural activity in individuals with IGD. Therefore, we need to include female subjects in the future to explore gender differences in static and dynamic brain local connectivity in IGD. Secondly, the sample size of this study was relatively small, future studies need to include more participants to verify the stability of this result. Thirdly, it is widely acknowledged that sleep disruption is closely linked with anxiety and depression. Depression and anxiety-related symptom scores for participants were lacking in this study. This information should be supplemented in future studies to explore the correlations between depression and anxiety and sleep quality in individuals with IGD. At last, because this study was cross-sectional, we couldn’t determine the causal relationship between addiction and abnormal brain activity. Longitudinal data are required to confirm and complement the current findings in future studies.

## Conclusions

In this study, we observed a wide range of aberrant intrinsic local connectivity in frontostriatothalamic circuitry in individuals with IGD, which may be related to the dysregulation between cognitive control and reward processing in IGD. In addition, the dReHo in the left caudate nucleus was correlated with PSQI scores in IGD subjects, which may indicate that the temporal variability of neural activity in the caudate nucleus may be a promising neurobiological marker linking IGD and sleep quality. These findings may provide new insights into the neuropathological mechanisms of IGD as well as the neural correlation between IGD and sleep quality.

### Electronic supplementary material

Below is the link to the electronic supplementary material.

Supplementary Material 1: Figure S1: Results at different window sizes showed significantly increased dynamic ReHo in IGD subjects compared to healthy controls.

## Data Availability

The datasets generated and/or analyzed during the current study are not publicly available due to confidentiality but are available from the corresponding author on reasonable request.

## List of abbreviations

IGD: internet gaming disorder
rs-fMRI: resting-state functional magnetic resonance imaging
IGDs: individuals with IGD
HCs: healthy controls
ReHo: regional homogeneity
sReHo: static ReHo
dReHo: dynamic ReHo
IAT: Internet Addiction Test
PSQI: Pittsburgh Sleep Quality Index
mSFG: medial superior frontal gyrus
SFG: superior frontal gyrus
SMA: supplementary motor area
DSM-5: Diagnostic and Statistical Manual of Mental Disorders, Fifth Edition
ICD-11: International Classification of Diseases, 11th Edition
BOLD: blood oxygen level dependent
KCC: Kendall’s coefficient of concordance
OFC: obitofrontal cortex
CBT: cognitive behavior therapy
pHIP: posterior hippocampus
rs-FC: resting-state functional connectivity
TR: repetition time
FOV: field of view
DPARSF: Data Processing Assistant for Resting-State fMRI
MNI: Montreal Neurological Institute
TDA: Temporal Dynamic Analysis
DPABI: Data Processing and Analysis of Brain Imaging
SD: standard deviation
CV: coefficient of variation
GLM: general linear model
FD: framewise displacement
ROC: receiver operating characteristic
AUC: area under the curve
DCM: dynamic causal modeling
PFC: prefrontal cortex
