# Supplementary figures and images for: Static and dynamic changes of intrinsic brain local connectivity in internet gaming disorder
Source: BMC Psychiatry. 2023 Aug 9;23:578. doi: 10.1186/s12888-023-05009-y (PMC10410779; doi:10.1186/s12888-023-05009-y)

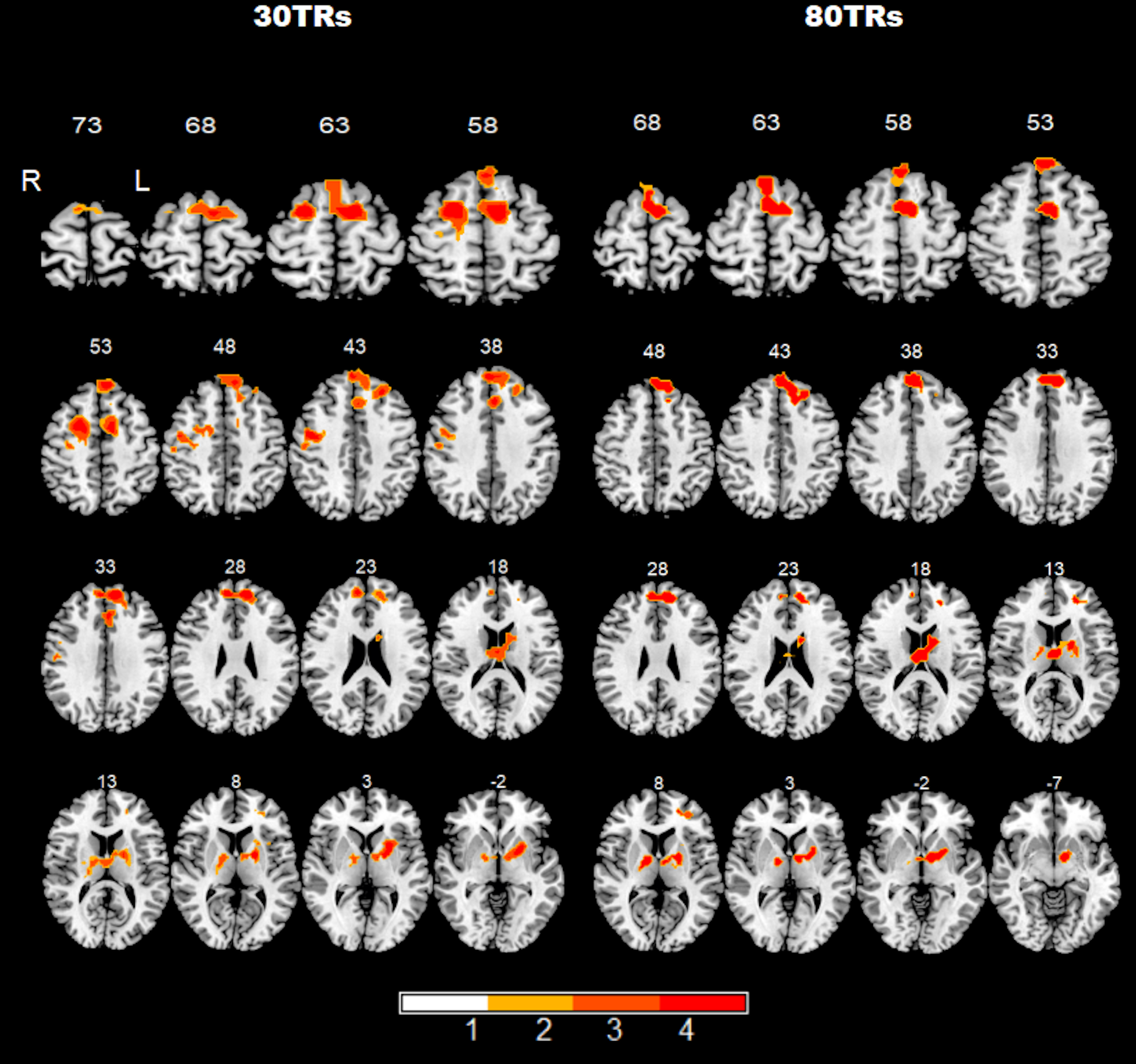

Supplement: Supplementary file 1 — Supplementary Material 1: Figure S1: Results at different window sizes showed significantly increased dynamic ReHo in IGD subjects compared to healthy controls. [file 12888_2023_5009_MOESM1_ESM.png]
